# Two-Year Clinical Outcomes of Patients Undergoing Synthetic Cartilage Arthroplasty for First Metatarsophalangeal Osteoarthritis

**DOI:** 10.7759/cureus.75683

**Published:** 2024-12-13

**Authors:** Abid Mahmood, Hussain Atcha, Shahnawaz Khan, Henry Atkinson

**Affiliations:** 1 Trauma and Orthopaedics, Maidstone and Tunbridge Wells NHS Trust, London, GBR; 2 Diabetes and Endocrinology, Essex Partnership University NHS Foundation Trust, Colchester, GBR; 3 Trauma and Orthopaedics, North Middlesex University Hospital NHS Trust, London, GBR

**Keywords:** cartiva, first metatarsophalangeal arthroplasty, foot & ankle surgery, hallux rigidus, synthetic cartilage implant

## Abstract

Background

Severe osteoarthritis (OA) of the first metatarsophalangeal joint (MTPJ) is usually treated by arthrodesis, which results in a loss of mobility in the joint. Cartiva (Stryker Corporation, Kalamazoo, USA) is a synthetic cartilage implant (SCI) designed to repair osteochondral defects in the treatment of the first MTP OA. Treatment using the Cartiva SCI should relieve symptoms of OA whilst sparing motion in the first MTPJ and may provide a superior alternative to arthrodesis.

Objectives

The objective of this article is to assess the clinical outcomes of a novel SCI, Cartiva, for arthroplasty in the management of the first MTPJ arthritis.

Study design and methods

We retrospectively reviewed 64 patients who had received a total of 67 Cartiva implants between May 2016 and June 2020. The average age of these patients at the time of surgery was 54.3 years with a range from 19 to 77 years. Forty-five of the 67 implants were used in females and 22 were used in males. Radiological Grade 2 OA was present in 46 cases with Grade 3 in 21 (Hattrup and Johnson classification). Validated patient-reported outcomes measures (PROMs) were used to assess the functional efficacy of the implants. All cases were performed by a single surgeon. The PROMs data was collected via follow-up phone calls with the patients who were asked questions from the American Academy of Orthopaedic Surgeons’ Foot and Ankle Measure (AAOS-FAM), the EQ-5D-3L survey and the Manchester-Oxford Foot and Ankle Questionnaire (MOXFQ). All data collected was anonymised to preserve confidentiality and local hospital ethics committee approval was sought. All statistical analysis was done using Microsoft Excel (Microsoft® Corp., Redmond, WA) and Statistical Package for the Social Sciences (IBM SPSS Statistics for Windows, IBM Corp., Version 23.0, Armonk, NY).

Results

There was a significant improvement in all PROMs at three years, and one patient developed an implant-related infection. The average scores for the EQ-5D-3L survey significantly improved from 0.69 to 0.85 post-operative (post-op) (p<0.05). The mean MOXFQ scores improved from 42.5 to 15.4 post-op (p<0.05). The radiologic evaluation demonstrated satisfactory implant positioning in all cases at the last follow-up.

Conclusions

The data shows significant improvements between pre-op and post-op scores. The implication of this is that synthetic cartilage arthroplasty provides a viable alternative to arthrodesis in the treatment of OA affecting the first MTPJ. However, more studies may need to be done focusing on larger sample sizes and patients should be observed over a longer term, including a formal multi-centred clinical trial.

## Introduction

Hallux rigidus (HR) is defined as osteoarthritis (OA) of the first metatarsophalangeal joint (MTPJ) and is recognised as the most common arthritic condition in the foot [1]. The mainstay of surgical treatment is arthrodesis, which involves fusion of the joint through internal fixation [2].

The main aim of arthrodesis is the resolution of pain whilst preserving a normal gait pattern [2]. However, arthrodesis is associated with significant complications including loss of motion, non-union (10%), metatarsalgia (10%), transfer lesions, and there is often a necessity for further surgery in more than 10% of patients [3,4]. The associated morbidity has led to alternative treatment options being sought to ameliorate MTP OA symptoms. Moreover, it is important to appreciate the role of the first MTPJ motion within the gait cycle particularly the "toe off" phase of the third rocker [5].

Synthetic cartilage arthroplasty involves replacing the damaged cartilage surface with a synthetic implant. Cartiva (Stryker Corporation, Kalamazoo, USA) is a synthetic hydrogel implant designed for the treatment of the first MTPJ OA to repair osteochondral defects [6,7]. Treatment using the Cartiva implant aims to relieve symptoms of OA whilst sparing mobility in the first MTPJ facilitating dorsi flexion, which has been shown to positively impact stride length and walking speed [8].

A prospective, randomised clinical trial conducted on behalf of the Cartiva motion study group concluded comparable pain relief and functional outcomes between first MTP arthrodesis and synthetic implant arthroplasty with follow-up at 12 and 24 months [7]. The study concluded that synthetic implants were a suitable alternative to arthrodesis for patients with prefer to maintain first MTPJ mobility. Brandao et al. conducted a prospective study of 55 patients undergoing Cartiva synthetic implant arthroplasty with an average follow-up of 21 months [9]. This series revealed statistically significant improvements in functional outcomes with low early revision rates in the short term.

Other alternatives to arthrodesis in the treatment of first MTPJ OA include silastic implants, which have been in use for over 50 years; however, these have traditionally been associated with high failure rates [10]. Newer generations of these implants have produced encouraging results although long-term data is scarce [10]. Many alternative implants using different materials are available including BioPro, MOJE and some three-component implants including Roto-Glide and ToeFit-Plus [11]. Outcomes of such interventions remain variable and insufficient data to support their utilisation.

Studies examining medium- to long-term outcomes of first MTP arthroplasty utilising the Cartiva synthetic cartilage implant (SCI) are scant. Longer-term outcomes of Cartiva have demonstrated high revision rates with a recent recall on the implant itself. The main objective study was to identify patient-reported outcomes at an average of 3.4 years in patients that undergo SCI arthroplasty for the first MTP OA from a high-volume fellowship-trained surgeon. Secondary outcomes assessed revision rates and complications. The study also seeks to re-frame the indications of this implant as a stopgap to arthrodesis.

## Materials and methods

We retrospectively reviewed 64 patients who underwent a total of 67 Cartiva implants between May 2016 and June 2020 with a minimum follow-up of two years via telephone. Data was prospectively collected. The average age of these patients at the time of surgery was 54.3 years (SD 12.9) with a range of 19 to 77 years (Table 1). There were 43 females who received 45 Cartiva implants and 21 males who received 22 implants. Validated patient-reported outcomes measures (PROMs) were used to assess the functional efficacy of the implants. All data was anonymised to preserve confidentiality.

**Table 1:** Demographic data of patients receiving Cartiva SCI arthroplasty SCI: synthetic cartilage implant; OA: osteoarthritis

Characteristics	Item	Count	Percentage (%)
Gender	Male	22	32.8
	Female	45	67.2
Age at time of surgery (years)	35 or below	5	7.5
	36 to 45	11	16.4
	46 to 55	18	26.9
	56 to 65	16	23.9
	66 to 75	16	23.9
	76 and above	1	1.5
Side	Right	39	58.2
	Left	28	41.8
OA radiographic severity	Grade 2	46	68.7
	Grade 3	21	31.3

Surgical technique

All procedures were conducted by a single fellowship-trained surgeon with over 10 years of experience in performing forefoot surgery. The procedures were done as day-case surgery under general anaesthetic for 30-40 minutes. The first MTPJ was approached medially and a cheilectomy was performed, leaving no sharp edges. The first metatarsal was then drilled into, and the implant was pushed in to leave a 2-3 mm edge around the joint surface. Post-operative care consisted of elevation for two weeks with a wedge shoe. Patients were given physiotherapy from two weeks post-operatively. They could return to work and drive from three weeks and play sports from six weeks after surgery.

Pre-operative radiological data on the severity of the first MTP OA was also collected with radiographic classification defined by Hattrup and Johnson, with Grade 1 showing mild OA with preserved joint space to Grade 3 with severe joint space narrowing, significant osteophyte formation, loose bodies, subchondral sclerosis and cysts [12]. OA of Grade 2 was present in 21 of the patients (31.3%) and 46 patients (68.6%) had Grade 3 OA.

Outcome evaluation

PROMs data was collected via follow-up phone calls with the patients who were asked questions from the American Academy of Orthopaedic Surgeons’ Foot and Ankle Measure (AAOS-FAM), the EQ-5D-3L survey and the Manchester-Oxford Foot and Ankle Questionnaire (MOXFQ). Data on complications and revisions was also collected. The AAOS-FAM consists of a 25-point questionnaire with five subscales that evaluate pain, function, stiffness and swelling, giving way and shoe comfort [13]. The AAOS-FAM results can be converted to produce scores from 0 to 100 for foot and ankle function where 100 represents the best outcomes. Additionally, the shoe comfort scale is also scored between 0 and 100 where 100 represents comfort in all shoe types.

The EQ-5D-3L provides a generic measure of health using five questions, which assess mobility, self-care, pain, usual activities and psychological status [14]. The results are applied to an algorithm, which produces a score between -0.59 and 1.00 where "1" represents perfect health. Additionally, the EQ-5D-3L questionnaire included a Visual Analogue Scale (VAS) in which patients were asked to provide a rating out of 100 of their current health state with 100 being the best health state they can imagine. The MOXFQ is a validated PROM designed to evaluate outcomes of foot or ankle surgery. It consists of a 16-point questionnaire with seven questions assessing walking or standing ability, five questions for pain and four questions to assess difficulties with social interaction [14]. The MOXFQ index consists of values between 0 and 100 where 100 indicates the worst outcomes. All statistical analysis was done using Microsoft Excel (Microsoft® Corp., Redmond, WA) and Statistical Package for the Social Sciences (IBM SPSS Statistics for Windows, IBM Corp., Version 23.0, Armonk, NY).

## Results

The mean follow-up of all patients was 3.4 years (2 to 5) post-operatively. Three out of 64 (4.7%) patients required bilateral surgery on separate occasions. The majority of patients reported no issues of mobility or pain in the target foot. Four (6.0%) out of the 67 implants required revision surgeries, giving an implant survivorship of 94% (63/67).

A total of 13 complications (19.4%) were reported with one patient developing osteomyelitis. The patient initially had Grade 2 OA and required revision surgery with removal of the implant, debridement and irrigation of the bone and a 16-week course of antibiotics. The joint developed progressive, painful degeneration and the patient underwent the first MTPJ fusion six months later.

Three additional revisions were carried out due to ongoing symptoms of pain and stiffness following the initial procedure with two out of three having Grade 3 OA and one having Grade 2 OA. They underwent conversion to the first MTPJ fusion at 14, 17 and 26 months post-operatively. Eight out of 67 cases (11.9%) reported ongoing pain post-procedure but none were severe enough to require revision. One patient had ongoing pain on straining but none at rest. Another patient experienced ongoing stiffness without pain (Table 2).

**Table 2:** Complications of synthetic cartilage implant arthroplasty in 67 cases

Complications	% (n)	Note
Ongoing pain	16.4 (11)	Three patients underwent further surgery for arthrodesis
Stiffness	1.49 (1)	Managed conservatively
Osteomyelitis	1.49 (1)	Required revision surgery and six weeks of antibiotics

**Figure 1:**
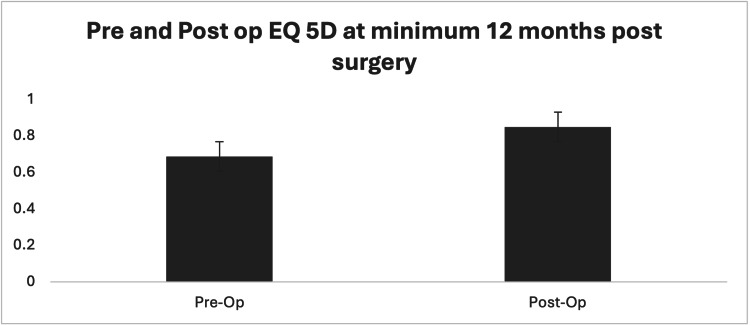
The EQ-5D index consists of values between 0 and 1, with "1" representing full health (P-value < 0.05).

Forty-eight out of 67 cases revealed significantly improved EQ-5D-3L scores at follow-up. The EQ-5D-3L revealed an average increase from 0.69 to 0.85 post-operatively with the largest improvements in raw scores for pain followed by the ability to carry out usual activities (Figure 1). The average VAS scores improved significantly from 71.8 pre-operatively to 83.8 (p<0.05). Paired t-test analysis of the pre- and post-operative EQ-5D-3L scores revealed a P-value of less than 0.05 indicating the difference to be statistically significant. The AAOS-FAM produced a mean core score of 86.01 and a mean shoe comfort score of 52.5.

**Figure 2:**
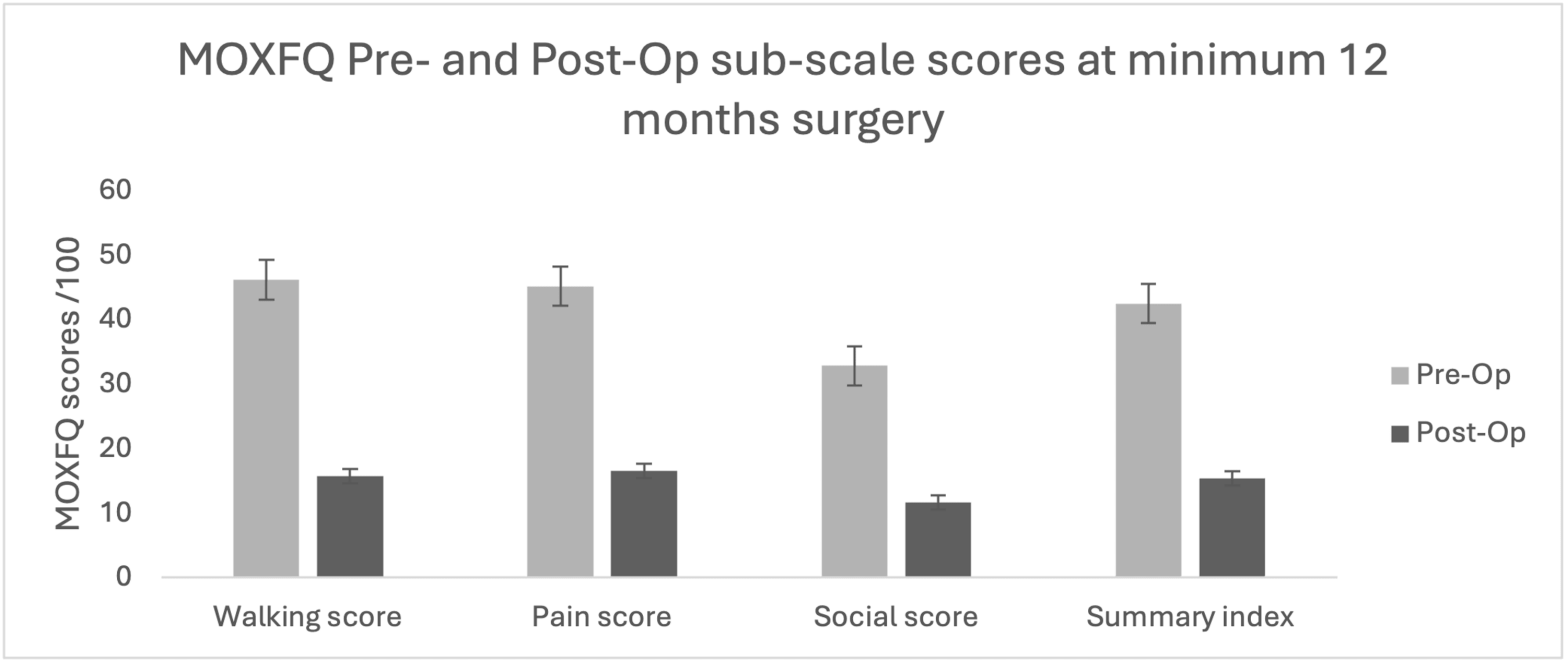
Graph illustrating the pre- and post-op MOXFQ sub-scale scores (lower scores indicate better foot function). MOXFQ: Manchester-Oxford Foot and Ankle Questionnaire

The mean MOXFQ summary scores improved from 42.5 to 15.4 post-operatively. Analysis of all MOXFQ subscales also revealed significant improvements. The greatest improvement was found in the mean walking score, which improved from 46.2 to 15.8 with the second largest subscale improvement in the mean pain score, which improved from 45.1 to 16.6 and the mean social score improved from 32.8 to 11.7. Paired T-test analysis of the pre- and post-operative MOXFQ summary scores revealed a P-value of less than 0.05 indicating the difference in the results to be significant (Figure 2).

## Discussion

The Cartiva SCI is a novel intervention in the management of the first MTPJ OA. It can negate the need for fusion of the joint. Compared to conventional arthrodesis, synthetic cartilage arthroplasty is less invasive and reduces alterations in gait mechanics. Studies outlining the efficacy of the implant are scant. This study demonstrates a significant improvement in patient-reported outcomes with minimal complications recorded. Improvements specifically in the MOXFQ scale can be attributed to increased joint mobility in comparison to pre-operative levels [15].

A similar study by Goldberg et al. directly compared the first MTP arthroplasty and arthrodesis and found no clinically significant differences between interventions although both cohorts improved in comparison to their pre-morbid state [16]. Males were found to have greater success with arthroplasty versus arthrodesis but statistical analysis of this was also not significant. It is noteworthy that patients with hallux valgus greater than 20 degrees were excluded. The findings of the current study reaffirm those of Goldberg et al. in outlining the efficacy and safety of SCI arthroplasty.

Baumhauer et al. compared SCI to a traditional arthrodesis approach and found a statistically significant reduction in VAS pain scores at 12 and 24 months post-operatively with the synthetic implant approach [5]. In addition to improved pain scores, the range of motion was also improved by 6.2 degrees, which was maintained two years post-implant. These findings represent the most desirable outcomes with the preservation of movement and a reduction in symptoms. The study concluded that synthetic arthroplasty was a better alternative to arthrodesis in patients who wished to maintain the motion of the first MTPJ. Arthrodesis sacrifices motion of the joint for alleviation of pain and this is often reported to have a significant impact on patients with gait and balance affected due to lateralisation of load in the operated foot to compensate for loss of first MTPJ mobility [17].

Brandao et al. showed statistically significant improvements in MOXFQ scores [9]. Summary scores dropped from 58 to 24 (34-point drop) similar to our study from 42.5 to 15.37 (37-point drop) [9]. Similarly mean walking score dropped from 58 to 25 (33-point drop) - our study revealed a 30.4-point drop reduction. Pain scores were also significantly improved. This is promising and shows that across separate studies Cartiva has demonstrated meaningful improvements for patients when using validated PROMs such as MOXFQ. Notably, Brandao et al. did report dissatisfaction amongst female users who desired to wear high-heeled shoes and found that even at 21 months follow-up, despite reduced pain and increased mobility, use of high-heeled shoes remained difficult to wear. However, this can be explained by the high degree of stress that high-heeled shoes place on the MTPJs [18].

Aynardi et al. researched the arthroplasty of the first MTPJ and found an overall failure rate of 3.8% (5/133) with five patients needing subsequent surgery [19]. This study did not mention the specific reasons for revision surgery (pain, infection and/or complications). However, with a similar failure rate to our study (three patients needed revision surgery), it shows promising results for Cartiva as a new treatment approach.

The traditional arthrodesis approach has complications such as non-union as well as pain and joint stiffness. A retrospective analysis by Prat et al. of 79 patients who underwent arthrodesis found the complication rate was 40.5% and 8.9% requiring further surgery, which carries yet further risk [20]. In comparison, our series had a much lower complication rate of 19.4%. In addition to Aynardi et al.’s study, this series shows a lower complication rate of subsequent surgery and further illustrates the potential promise of synthetic implant approaches.

Silastic implants are an existing alternative to arthrodesis in the treatment of the first MTPJ OA and have been in use for over 50 years [10]. Initial iterations of silastic implants were subject to high complication rates and early failure leading to revision; however, subsequent generations with a double-stemmed rather than a single-stemmed implant have shown encouraging results. Sethi et al. conducted a retrospective analysis of 117 silastic arthroplasties treating end-stage HR and found a complication rate of 8.9% with only one patient requiring revision surgery at an average follow-up of four years [11]. These encouraging results further illustrate the first MTPJ arthroplasty as a viable alternative to arthrodesis. As with our study, these results are limited to the medium term and data was collected retrospectively.

The BioPro metallic hemiarthroplasty implants were introduced over 60 years ago [21]. Clement et al. reported a 14.4% revision rate in the medium term in a retrospective review of 97 patients who underwent a metallic hemiarthroplasty procedure [21]. The authors associated the high revision rate with younger patients and concluded the BioPro implant is a useful short to mid-term measure to improve function in older patients suffering from HR. Given the lower revision rates reported in our study, Cartiva SCI may prove a superior short- to medium-term intervention compared to the BioPro implant. The ceramic prosthesis, MOJE, is another implant that was first used in the early-2000s [22]. Despite favourable initial outcomes, Dawson-Bowling et al. reported a reoperation rate of 26% at an average follow-up of 6.7 years with 52% of implants loosening [22]. Three-component implants such as Roto-glide have shown promising short-term results in the management of HR, however, longer-term data is scarce [23]. Another metallic multi-component implant, ToeFit-Plus, has shown very high reoperation rates in the mid-term [24].

Our study does demonstrate a rate of 16.4% (n=11) of patients with ongoing pain with three of these patients requiring further surgery. Closer analysis of demographic data reveals six out of 11 patients were female and five were male. The average age of these 11 patients at the time of surgery was 56.9 years with a range of 37 to 73 years. All three patients requiring further surgery for pain were males with an average of 60.7 years at surgery. Further research will be required to identify significant common demographic qualities in HR patients who continue to have pain following SCI arthroplasty. Cartiva may represent an interim measure in patients requiring delayed fusion within this cohort.

This is already evident in many cases in the United States as the Cartiva SCI has been subject to significant scrutiny due to higher-than-expected revision rates. Radcliffe and Roukis reviewed 236 individual reports of adverse events documented in relation to the Cartiva SCI over an 84-month period and found implant removal occurred in 73.7% of these cases at the time of publication, with 67.7% of 102 revision surgeries resulting in the first MTPJ fusion [25]. The authors attributed this revision rate to the sparsity of published data on medium- to long-term outcomes in patients undergoing Cartiva SCI arthroplasty. Given this study reports a 6.0% revision rate at a mean follow-up of 40 months, further research is indicated to identify the patient demographics that would most benefit from SCI arthroplasty. Given the safety profile demonstrated withdrawal of this intervention will represent a diminution in surgical options for managing MTPJ OA.

A study by Partio et al. of 18 patients who underwent a synthetic arthroplasty concluded that a statistically significant improvement in VAS scores from 10 to 77.5 was seen but also mentioned that two patients only experienced temporary relief of pain [26]. Both these patients went on to have revision arthrodesis surgery of the first MTPJ. Despite this, Partio et al.’s work is promising and again highlights the statistically significant benefit of synthetic implant approaches for patients. Pain levels are markedly improved, and complication rates remain minimal. This adds to the potential promise and efficacy of synthetic implants to become the new mainstay of treatment, with arthrodesis reserved for patients who suffer post-operative complications such as ongoing pain. The reasons why a small percentage of patients do experience ongoing pain symptoms despite synthetic implant arthroplasty remain unclear and other factors such as severity of OA pre-operatively, BMI index and other disease burdens could be contributing. Further research will need to closely examine the reasons for post-operative pain and complications and if these can be mitigated with lifestyle or medical management then the surgical efficacy of synthetic implants can be further supported versus traditional arthrodesis.

One patient in our study developed osteomyelitis requiring a prolonged course of antibiotics. Whilst only one patient experienced this complication, it is still an important consideration as a possible complication of an arthroplasty procedure. The conditions surrounding this case and the development of osteomyelitis were not investigated but potential factors to consider in future are improved sterility in the operating theatre as well as protection of the synthetic implant before it is surgically implanted.

The study is limited by a small sample size of only 64 patients. A future consideration for this study would be to include a larger patient size over a longer period. This would produce more reliable data over a longer timeframe demonstrating immediate, short and long-term outcomes of Cartiva SCI arthroplasty. Synthetic implants are a novel approach to the treatment of first MTP OA and it is worth noting that there is little literature currently on its efficacy as shown by Smyth et al. [27]. Further studies would provide valuable insights into synthetic implants and given the dwindling literature on the topic, would prove extremely useful for clinical outcomes.

## Conclusions

This study does show significant benefits of first MTP Cartiva arthroplasty with reduced pain, greater range of motion of the joint and thereby improved mobility. Overall, the data shows significant improvements between pre- and post-operative PROMs with minimal complication rates. Therefore, there is real potential and promise for synthetic arthroplasty using Cartiva to become the new gold standard for the first MTP OA treatment. However, more studies may need to be done focusing on larger sample sizes and patients should be observed over a longer term, including a formal clinical trial.

Irrespective of the encouraging results, it is important to note that the implant has been subject to intense scrutiny due to higher-than-expected revision rates. In part, this is due to limited published long-term data regarding its efficacy. This paper advocates the use of this implant as a "stop-gap" between fusion and conservative management to maintain a more physiological range of motion.
